# Oral health in transition: The Hadza foragers of Tanzania

**DOI:** 10.1371/journal.pone.0172197

**Published:** 2017-03-15

**Authors:** Alyssa N. Crittenden, John Sorrentino, Sheniz A. Moonie, Mika Peterson, Audax Mabulla, Peter S. Ungar

**Affiliations:** 1 Metabolism, Anthropometry, and Nutrition Laboratory, Department of Anthropology, University of Nevada, Las Vegas, Nevada, United States of America; 2 D.M.D. Family and Cosmetic Dentistry, New York, United States of America; 3 Epidemiology and Biostatistics, School of Community Health Sciences, University of Nevada, Las Vegas, Nevada, United States of America; 4 Dorobo Fund, Arusha, Tanzania; 5 Department of Archaeology, University of Dar es Salaam, Dar es Salaam, Tanzania; 6 Department of Anthropology, University of Arkansas, Fayetteville, Arkansas, United States of America; Max Planck Institute for the Science of Human History, GERMANY

## Abstract

Conventional wisdom holds that a decline in oral health accompanies the transition from hunting and gathering to agriculture, given increased consumption of carbohydrates. This widely touted example of the mismatch between our biology and modern lifestyle has been intuited largely from the bioarchaeological record of the Neolithic Revolution in the New World. Recent studies of other populations have, however, challenged the universality of this assertion. Here, we present the first comprehensive study of oral health among a living population in transition from the bush to village life, the Hadza hunter-gatherers of Tanzania, to test the hypothesis that the shift from foraging to farming, or agricultural intensification, inevitably leads to increased periodontal disease, caries, and orthodontic disorders. Our results showed that women living in villages consuming a mostly agricultural diet exhibited more caries and periodontal disease than those living in the bush consuming a mostly wild-food diet. Furthermore, men living in the bush consuming mostly a wild-food diet had more than those living in the village consuming a mostly agricultural diet. These findings are explained by the high incidence of maize consumption in village settings, along with previously recognized variation in rate of caries between men and women. The unexpected discovery of high caries incidences for men in the bush is likely explained by heavy reliance on honey, and perhaps differential access to tobacco and marijuana. These data support the notions that mechanisms of cariogenesis are multifactorial and that the relationships between oral health and the shift from a predominantly wild-food diet to one dominated by cultigens are nuanced.

## Introduction

The transition from hunting and gathering to agriculture is routinely associated with declines in oral health, given increased consumption of carbohydrates and growth of bacterial colonies in dental plaque linked with the development of dental caries (e.g. *Streptococcus mutans* and other associated bacteria) and periodontal disease. The most widely held hypothesis suggests that dental caries form as tooth enamel and dentin are demineralized by acids produced from plaque bacteria during the fermentation of carbohydrates [1]. Further, long-standing colonies of plaque bacteria provoke an immune response resulting in inflammation of gingival tissues, disruption of the periodontal ligament, resorption of the alveolar bone and, ultimately, tooth loss [2]. Indeed, it has been argued that the “amount of carbohydrates consumed and reliance on domesticated plants largely explains the variation in prevalence of dental caries in archaeological and other settings” [3:68].

While diet may be the “main driver” of dental decay [4], a number of other factors that may influence the development of caries have been identified. These include the individual’s oral microbiome [5], feeding periodicity [6,7], geochemistry of the oral environment [1,8], rate of dental wear [9], genetic susceptibility [10], the oral health of the mother [11], and the link between saliva flow, salivary biochemistry, female hormones, and life history events [12, 13, 14], among others.

Dental diseases have been particularly important to anthropologists in studies of bioarchaeology and paleopathology, largely because teeth are resistant to degradation compared to soft tissues [2, 3]. While caries are common today, they have only been documented in approximately 2% of observed Paleolithic and Mesolithic populations [15], suggesting that, at least in some parts of the world, rates have increased significantly with the onset and intensification of agriculture [16, 17]. Indeed, Turner [18] suggested a five-fold increase in caries frequency with the Neolithic revolution. This is typically explained as a mismatch between oral biology and the dietary environment in which teeth and gums evolved.

That said, the effect of agriculture on caries incidence varied across the globe [6,19], and was dependent upon the cereal staple consumed (e.g., maize, rice, wheat, etc.). Caries rate clearly climbs with maize consumption [3, 20, 21], though European and western Asian Neolithic populations reliant on wheat did not show the same increase [22, 23]. In fact, there is no clear increase in the Neolithic caries rate from Natufian to the Neolithic in the Levant. Asian populations consuming rice also have low caries rates compared with maize-based populations, and in some cases, there was actually a decline in caries with the transition from foraging to farming [24].

Indeed, some hunter-gatherers had rather high caries rates related to carbohydrate-rich, and especially sugar-rich, diets. Mesolithic peoples from Sicily and Portugal, for example consumed cariogenic foods such as honey, dates, and figs [23, 25]. Similarly, prehistoric foragers from Northwest Mexico also have high rates of dental caries due to their reliance on cactus, another highly cariogenic food [26]. Indeed, oral health data from recent [27, 28, 29, 30] and prehistoric [26, 31, 32] foragers provide mounting evidence of periodontal disorders, high prevalence of caries, and antemortem tooth loss among hunters and gatherers.

To date, however, few studies have documented oral health among living small-scale populations [33, 34]. Here, we provide the first data on oral health among the Hadza peoples of Tanzania. As different groups of Hadza foragers are regularly consuming diets with varying proportions of agricultural products and wild foods, data from the Hadza provide a unique opportunity to observe directly the effects of a transition from bush-dominated to cultigen-dominated diets on oral biology and health in a single population.

### Study population

The Hadza reside in a 4000km^2^ area around the shores of Lake Eyasi in Northern Tanzania, East Africa. The total population size, approximately 1000 individuals, has shown no major disruption during the past 100 years. Only around 150 individuals, however, currently practice a predominantly hunting and gathering way of life, meaning that the bulk of their diet is derived from wild plant foods and game animals [35]. While no human population in Africa relies exclusively on wild foods any longer, these remaining bush-dwelling Hadza consume few cultigens. This makes them a valuable population with which to study effects of a shift in diet on oral health because, based on current demographic estimates, around 15% of individuals in Hadza camps choose a sedentized lifestyle more reliant on cultigens each year [36]. The current study is the first published on oral health among the Hadza—where bush-dwelling individuals rely on a primarily wild-food diet, villagers consume mostly agricultural products, and those that split their time between bush and village camps have mixed diets, intermediate in proportion of cultigens.

### Diet composition

Hadza living in the bush consume a diverse diet composed of a wide variety of plant foods, a vast array of bird species, small- to large-size game, and the larvae and honey of both stingless and stinging bees [36]. Plant products include baobab fruit, several types of berries, figs, drupes, legumes, and several species of tubers (underground storage organs). Animal products include honey and larvae (from both stinging and stingless bees) and a wide array of avian and mammalian meat—ranging from small to large game animals. For a detailed consideration of the specific wild foods that comprise the Hadza bush diet, see S1 Text.

Only a handful of quantitative studies of diet composition are available, yet they have remained consistent over the past 20 years in the estimated contribution of particular food types to the Hadza bush diet [36]. While the bush estimates for agricultural products are low, it must be noted that no Hadza are thought to regularly consume a diet that is composed of 100% wild foods, as those residing in the bush have at least occasional access to traded cultigens [37]. The two most recent detailed estimates of Hadza bush diet (collected in the same bush camps as the current study) combine to provide a breakdown in kilocalorie contribution of approximately: 46–53% wild-foraged plant food, 11–15% honey and larvae, 32–35% hunted avian and game meat, and 0–8% agricultural products [35, 38].

Over the past two decades, the introduction of ethnotourism has greatly increased the number of Hadza who live in or near villages, where individuals make a living by taking tourists on short daily hunting and gathering treks and performing Hadza songs and dances [39]. Others may work part-time as guides for game hunting safari companies, as paid guards for the farms of neighboring tribes, or as seasonal employees of ethnotourism companies, researchers, missionaries, or non-government aid organizations (NGOs). The Hadza living in and around villages practice a mixed subsistence regime and supplement bush foods with traded, purchased, and/or donated foods. The wild bush foods targeted by men in village camps are severely limited, compared to bush camps, and include some game animals such as birds, squirrels, dik dik, baboon, and hyrax. Wild bush foods targeted by women include primarily fruits, such as baobab (*Adansonia digitata*), palm fruit (*Hyphaene petersiana*), and available berry species (e.g. *Cordia sinensis*). Women have also been reported to collect “wild herbs”, including *Potolaka olarecea*, *Ceratotheca sesamoides*, and *Limeum viscosum*, typically found on the old settlement sites of the neighboring pastoralists [40]. The donated foods are typically rice, maize, and beans and tend to come from non-profit aid organizations and missionaries, with food donation drop offs occurring approximately every two months (as documented by author MP). Rice and maize are staple resources for the village populations in the surrounding areas and the consumption of home -made maize alcohol is quite common in the region [40, 41]. The purchased/traded foods include onions, avocadoes, amaranth greens (known in Swahili as “mchicha”), and domesticated meat (chicken, goat, or beef).

## Materials and methods

### Ethics statement

Human Research Subjects approval was obtained from both the University of Nevada, Las Vegas Institutional Review Board (IRB) in the office of Research Integrity and the University of Arkansas Office of Research Compliance. Informed consent was obtained orally from all participants, as the Hadza are a non-literate population. Participant consent was recorded and witnessed affidavits were obtained. The IRB offices at both universities and the necessary Tanzanian government agencies approved the consent procedure. All data were collected with the permission of the Tanzanian Commission for Science and Technology (COSTECH) and the Tanzanian National Institute for Medical Research (NIMR).

### Individuals studied

A total of 75 adult individuals (37 women; 38 men), age 18 years or more, were included in this study. These included people residing in bush camps located on Gideru Ridge (*Buluku*, *Sangeli*, and *Kisanakwipi*) and those living in camps located within the village system of Mangola (*Manudu*, *Mandagau*, *Maweni*, *No*!*Na* and *Tsiorobe*). All data were collected over a total of ten days in July of 2015. An effort was made to achieve a balanced design of males and females residing in the bush (consuming a largely wild-food diet), those in the village, and transients that moved regularly between the two locales, corresponding to those consuming low, high, and medium agricultural diets respectively.

### Residence data collection

For each individual, name, sex, and age were recorded (only sex and age are reported in S1 Table). Age estimates were determined using long-term demographic data collected over the last 10 years by author ANC and over the last 40 years by anthropologists Frank Marlowe and Nicholas Blurton Jones. Ages of unknown individuals were estimated by ANC based on interview data of camp members and relative age to known individuals from the census. Our sample included only adults over the age of 18 years, per UNLV and University of Arkansas Human Research Subjects protocol. Sample size was further limited due to geographic limitations and distance between bush camps.

Based on interview responses, all participants were binned into one of the following three residential groups, which we used as a proxy for diet composition: (1) bush (2) intermediate, and (3) village. The category of “bush” is a proxy for a diet low in agricultural products; the category “intermediate” is a proxy for a diet that is variable in agricultural products (with an intermediate proportion of cultigens over a long timeframe); and the category “village” is a proxy for a diet that is high in agricultural products.

While an exhaustive list of all camps each individual ever resided in would be impossible to confirm, a series of general questions did allow us to characterize the overall adult pattern of residence and diet. Our questions were similar to those asked by Frank Marlowe [36: p.41]. While participants’ recollections of their specific residence and diet over a lifetime may be imperfect, it was important to obtain a general idea of long-term diet—which is linked to caries formation and periodontal health. Therefore, each participant was asked the following questions (which correspond to Q2-Q7 in the supporting information S1 Table, with identifying information provided in Q1 removed):

1. What is the name of the camp where you were born?
2. Is that camp located in the bush or near a village?
3. When you were a child, would you say that you lived most of your childhood in the bush, in the village, or going back and forth between the two?
4. Would you say that during your childhood you at mostly bush foods, village foods, or both?
5. Would you say that for most of your adult life you have lived in the bush, in the village, or going back and forth between the two?
6. Would you say that for most of your adult life you have you eaten mostly bush foods, village foods, or both?
7. Do you go to the village often?

Each participant was binned into their respective groups (i.e. “bush”, “intermediate”, or “village”) in the field by ANC and MP based on the responses to the interview questions. All oral health measures were scored by JS later in a blind study (see below), without knowledge of group affiliation for any participant.

### Oral health data collection (field)

Oral health data were collected from individuals participating in the study (n = 75) using the procedure outlined below. While the total sample size is 75 individuals, some individuals were missing teeth in some positions, precluding them from participating in all orthodontic measurements. When the sample size is less than 75, it is noted in the texts. The participant first cleaned teeth for approximately two minutes using a pre-pasted toothbrush, and then rinsed with water as necessary. Fissures in teeth were further cleaned with a dental sickle scaler by PSU prior to study as necessary to remove tartar to allow visualization and laser fluorescence analysis of the crown.

Initial oral health data were collected by PSU, following training in a clinical setting with licensed clinical dentist, JS at his practice in New York. Dental photographs (occlusal and buccal views of all teeth), periodontal pocket depths, and enamel mineral density values were recorded for each individual. Presence of periodontitis, dental crowding (including malocclusion and third molar occlusion), number of identifiable caries, and gross wear were all assessed from photographs following return from the field by JS.

#### Oral photography

A standard suite of oral photographs was taken with a 16 Mp Olympus digital camera modified for intraoral photography (Lester A. Dine, Inc). Cheek retractors and intra-oral mirrors were used following convention for clinical dental photography. A total of nine images were taken of each subject: anterior, right buccal and left buccal views of teeth in occlusion, and upper and lower, left and right cheek teeth and anterior teeth in occlusal view. This allowed assessment of premolar and molar occlusal surface caries.

#### Periodontal probing

A calibrated standard Michigan O Probe with Williams markings periodontal probe was placed in the recesses around the C_1_ and M_1_ of each individual. Clinical experience suggests that recesses around these teeth are excellent proxies for overall periodontal health. Periodontitis score was calculated as the summed depth in millimeters of the six recesses (mesiobuccal, buccal, distobuccal, mesiolingual, lingual, and distolingual) around each tooth.

#### Laser fluorescence

Laser fluorescence of crown enamel was measured using a battery operated DIAGNOdent Classic Laser probe (KaVo). This instrument is designed to detect incipient caries. Occlusal surfaces of all cheek teeth (R+LP_3_-M_3_, R+LP^3^-M^3^, corresponding to numbers 1–5, 12–21, and 28–32 in the universal numbering system) were scanned, focusing on fissures, and points with fluorescence values exceeding 30 (the threshold value for demineralization reported by the manufacturer) were recorded and mapped on a dental chart for each individual.

### Oral health data collection (laboratory)

The combination of dental photographs, periodontal pocket measures, and laser fluorescence allowed us to characterize and compare multiple aspects of oral health. For each participant, the following measures were scored and recorded.

#### Orthodontics

Orthodontic condition was documented using several criteria. These included:

- Anterior dental crowding (Crowd Measure). This was measured on dental photographs as the ratio of the summed mesiodistal lengths of the occlusal surfaces of all lower anterior teeth (R+LI_1_-C_1,_ corresponding to numbers 6–11 and 22–27 in the universal numbering system) divided by the straight-line distance between the distal edges of RC_1_ and LC_1_ crown tips. When teeth were present, crowding was also scored using a 0–3 scale (Crowd Code): 0) no crowding; 1) minimal crowding; 2) moderate crowding, and 3) severe crowding.
- Angle’s Classification of malocclusion. This was assessed from photographs using Mosby’s Medical Dictionary [42] classes (http://medical-dictionary.thefreedictionary.com/Angle’s+classification+of+malocclusion): 1) neutrocclusion, where the “mesiobuccal cusp of the permanent maxillary first molar occludes in the buccal groove of the permanent mandibular first molar”; 2) distocclusion, where the “mesiobuccal cusp of the permanent maxillary first molar occludes mesial to the buccal groove of the permanent mandibular first molar”; and 3) mesiocclusion, where the “mesiobuccal cusp of the permanent maxillary first molar occludes distal to the buccal groove of the permanent mandibular first molar”.
- Third molar occlusion. This was scored from photographs as: 1) no M3s erupted; 2) one or more M3 erupted, but not in occlusion; and 3) all M3s in occlusion.
- Gross Wear. This was scored for the LM^1^ and LM^2^ of each individual using the following coding scheme: 0) unworn; 1) facet development with slight dentin exposure; and 2) essentially flat surface with considerable enamel loss and exposed dentin. Values were summed for the two teeth. This was used in lieu of more traditional techniques [43] to assure sufficient samples in each wear category for analysis.

#### Caries

Caries were scored using two methods: (1) visual identification in the field by PSU and ANC, and diagnostic confirmation using photographs in the laboratory by JS; and (2) using the DIAGNOdent Classic dental laser in the field by PSU with corroboration by JS in the lab using photographs.

Visual inspection entailed documenting each affected occlusal surface on the molars and premolar teeth, yielding a decayed, missing, and filled surfaces (DMFS) index [44]. Occlusal carious lesions were then scored by type, as follows by number of surfaces affected (1, 2, 3, 4, or entire tooth). The severity of the lesions were scored as follows: 1) incipient decay (i.e. detected by the dental laser but not obviously visible in the field); 2) obvious visual decay but no pulpal exposure; and 3) pulpal exposure. The visual inspection allowed us to calculate presence or absence of caries and an aggregate number of visible caries for each individual (CariesVis) divided by the number of cheek teeth in each mouth.

The DIAGNOdent values recorded in the field and their locations on the dental charts were compared by JS against the digital photographs in the lab. This allowed us to check for ‘false positives’ that might have occurred due to staining, tartar, or dentin exposure. Visible caries were confirmed in all cases with high DIAGNOdent values. When both values were recorded, only one was counted. In all cases where a carie was seen in the field, it was obvious also on the photographs; there were no false positives. The aggregate total number of caries for each individual was calculated (CariesAll) by combining the DIAGNOdent data with the visual inspection data divided by the total number of cheek teeth in each mouth.

#### Periodontitis

In addition to aggregate periodontal pocket depth, periodontal condition was scored according to a 0–4 for PerioPast and PerioPresent: 0) no evidence of periodontitis; 1) evidence for mild periodontitis; 2) evidence of moderate periodontitis; 3) evidence of severe periodontitis. PerioPast was visible evidence of past periodontal disease, whereas PerioPresent was visible evidence of periodontal disease at the time of measurement.

### Statistical analyses

Tests for differences in proportion between groups were conducted using the Pearson’s chi squared test or Fisher’s exact, given small sample sizes. T-tests, ANOVAs and correlation coefficients (or the nonparametric equivalents) were used to identify mean differences between groups and correlations between variables. Logistic, linear and multinomial regression were used to test for main and interaction effects.

When the categorical dependent variables had greater than two categories (Gross Wear, PerioPast, and PerioPresent) a point-biserial (PB) correlation [45,46], as a special case of Pearson’s product moment correlation, was used—with all results confirmed by Kendall’s Tau. When the sample size was small (<10 cases in a cell), Fisher’s exact test was used. In order to determine the interaction effects between sex, diet, and the orthodontic measurement of interest, age was controlled for and linear, binary, and multinomial logistic regression models were performed using forced entry for the main effects and stepwise entry, forward method, for the interaction between sex x location. For models with binary outcomes, logistic regression was used. For models with continuous (discrete) outcomes, linear regression was used. For models with 3 or more ordinal discrete levels, multinomial logistic regression was used.

### Results

Responses to the interviews allowed us to bin each participant into one of the following three groups: (1) bush (2) intermediate, and (3) village. The bush group (n = 25; 12 women, 13 men) consisted of people residing in bush camps located on the Gideru Ridge, (*Buluku*, *Sangeli*, and *Kisanakwipi)* where the majority of diet was composed of wild foods. The village group (n = 25; 19 women, 6 men) consisted of people residing in camps located within the village system of Mangola, (*Manudu*, *Mandagau*, *Maweni*, *No*!*Na* and *Tsiorobe*) where diet was mostly composed of domesticated foods. The intermediate group (n = 25; 7 women; 18 men) consisted of people who characterized the their adulthood residence as generally split between bush camps and village camps, consuming a diet dominated by neither wild nor cultivated foods.

#### Anterior dental crowding

A total of 65 individuals had enough teeth to assess anterior dental crowding.

No significant differences were found across ages (Pearson’s PB p = 0.420), between the sexes (Fisher’s Exact p = .098), or between groups (Fisher’s Exact p = 0.767). The interaction effect for the regression model of sex x location for Crowd Measure was also not significant (Chi-square = 5.563; p = 0.936).

#### Angle’s classification of malocclusion

**Fig 1:**
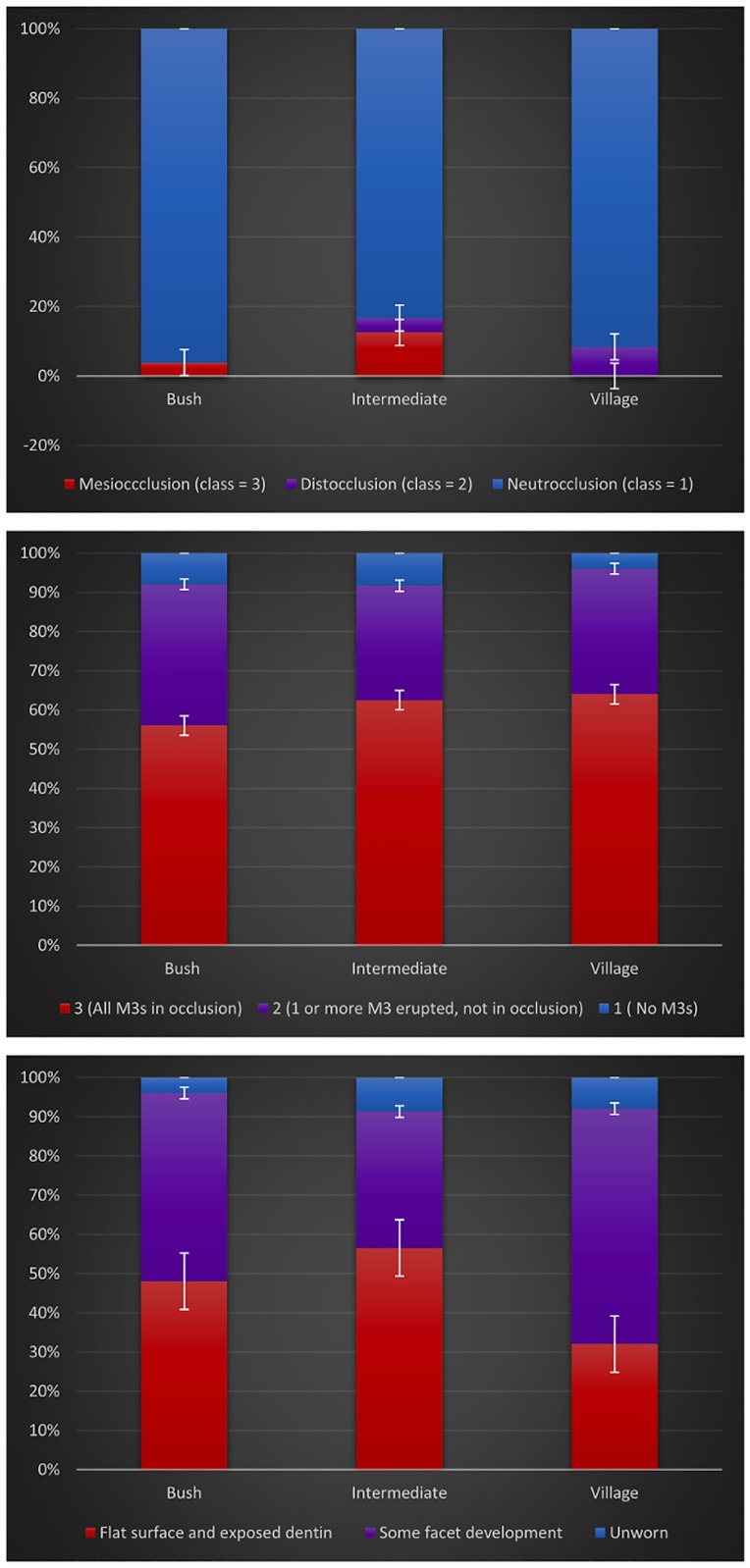
(a) Angle’s classification; (b) third molar occlusion; and (c) gross wear.

A total of 74 individuals were assessed by Angle’s Classification of malocclusion. Across all groups, the overwhelming majority (89%) of individuals were classified with neutrocclusion (Class 1). See Fig 1. Of the remaining individuals, 4% were classified with distoclussion (Class 2) and 5% evinced mesiocclussion (Class 3). Due to the unbalanced sample size (68 individuals fell into the category of neutrocclusion), we were unable to perform analyses in a statistical model testing the interaction effects of sex, age, or location.

#### Third molar occlusion

A total of 74 individuals had the presence and/or eruption of the third molar scored. Across all groups, the majority (61%) had a score of 3 (all M3s in and in occlusion), 32% had a score of 2 (one or more M3 erupted, but not in occlusion), and only 7% had a score of 1 (no M3s). See Fig 1. No significant differences were found across ages (Pearson’s PB p = 0.364), between sexes (Chi-square = 0.279; p = 0.598), or between residential groups (Chi-square = 0.368; p = 0.828). The interaction effect for the regression model of sex x location for M3 Occlude was also not significant (Chi-square = 2.924; p = 0.818).

#### Gross wear

A total of 73 individuals had enough teeth present to measure gross wear. Across all residential groups, 45% exhibited severe wear, wherein the molars measured (LM^1^ and LM^2^) had flat surfaces with exposed dentin, 48% exhibited moderate wear with some facet development, and 7% showed no evident gross wear (Fig 1). Increase in age corresponded with increase in gross wear across all groups (Pearson’s PB p<0.001; Kendall’s Tau p<0.001). Controlling for age, no significant differences in gross wear patterns were found between sexes (Chi-square = 2.117; p = 0.549) or by location (Fisher’s Exact p = 0.5864). There was no significant interaction for the model sex x location for Gross Wear, p = 0.355).

#### Missing, broken, and carious teeth

**Fig 2:**
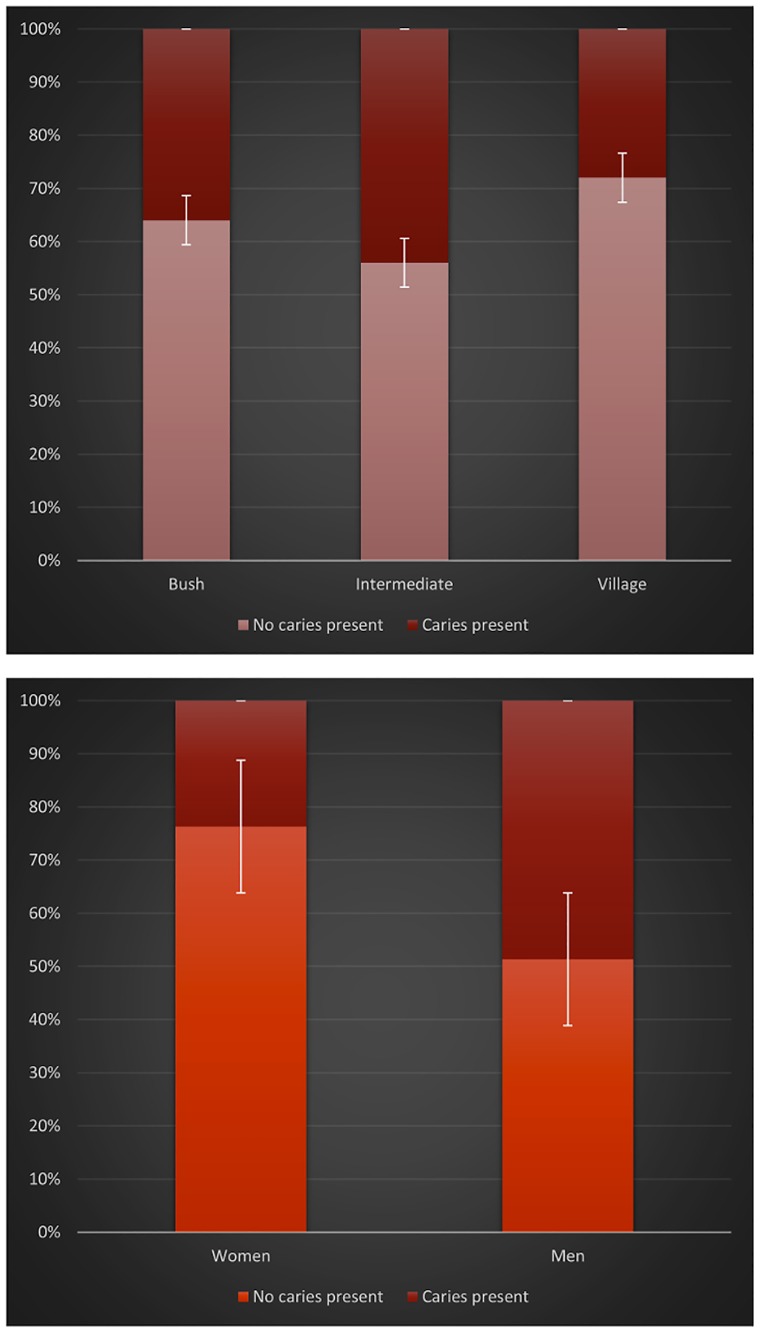
Percentage of carious teeth by (a) location (b) sex.

A total of 23 individuals had missing or broken teeth, with the highest rates of missing/broken teeth in men and those living in the bush with a low agricultural diet. A total of 75 individuals had enough teeth present to measure rate of caries. Across all groups, 36% (24% of women and 49% of men) had at least one visible carious lesion (Caries Vis) (see Fig 2). No significant differences were found between residential groups (Chi-square = 1.389; p = 0.499), yet significant differences were found between the sexes, with men exhibiting more visible caries (Chi-square = 5.071; p = 0.024). The aggregate total number of caries for each individual was also calculated (Caries All) for a total of 75 individuals, combining the scoring from the DIAGNOdent dental laser with the visual inspection data (Caries Vis). Across all groups, increase in age corresponded with increase in dental decay (Pearson’s p = 0.043).

**Fig 3:**
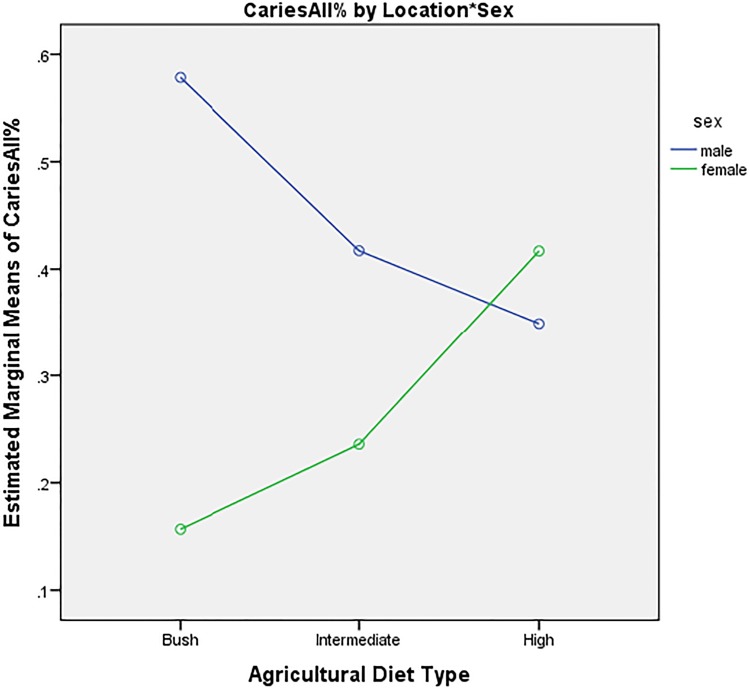
Prevalence of caries by sex, stratified by location.

The interaction effect model for sex x location for Caries All was significant (t = 3.693, p <0.001) (β = 0.48). Males living in the bush consuming a diet with few cultigens show a significantly higher caries incidence than those living in the village consuming a high agricultural diet (t = -3.444, p = 0.001) (β = -0.26). Splitting time between the bush and the village was not a significant predictor for increased or decreased caries for men (t = -1.820, p = 0.73) (β = -0.17). Women living in the bush and consuming a low agricultural diet were at significantly lower risk of caries than those living in the village (t = -3.693, p = 0.042) (β = 0.21). Splitting time between the bush and village (intermediate category) was also not associated with increased or decreased caries prevalence for women (t = -1.705, p = 0.93) (β = -0.23). See Fig 3.

#### Periodontitis

A total of 75 individuals had enough teeth present to assess periodontitis in the past and at the time of measurement. Across all groups, 66% of individuals showed evidence of periodontitis in the past (i.e. mild, moderate, or severe) and 80% showed some degree of periodontitis at the time of measurement. A significant correlation was found between increase in age and increase in degree of periodontitis for PerioPast (Pearson’s PB p<0.001; Kendall’s Tau p<0.001) and PerioPresent (Pearson PB p<0.002; Kendall’s Tau p < 0.001). Across the entire sample (bush, intermediate, and village camps), women showed significantly lower rates of PerioPast (Chi-square = 13.199; p = 0.001) and PerioPresent (Chi-square = 12.959; p = 0.002) when compared to men. Residential setting alone, however (bush, intermediate, village), was not a significant predictor of PerioPast (Chi-square = 7.077; p = 0.132) or PerioPresent (Fisher’s Exact p = 0.2558).

### Discussion and conclusions

Despite differing diets, Hadza foragers living in the bush and the village both show evidence of caries, periodontal disease, and gross wear. Not surprisingly, a significant increase in age mapped onto all of these orthodontic measures, which supports the findings of previous studies on oral health among small-scale societies [47, 48, 49], including rural populations in Tanzania [50]. No significant degree of dental crowding or third molar occlusion were found in any residential group, between sexes, or across ages, which also is also consistent with findings of previous studies of some populations outside of the post-industrialized west [29, 51, 52, 53], including rural Tanzania [54]—but for contradictory results concerning M3 occlusion, see [55].

We did not find any notable differences in gross wear patterns between the sexes, though different teeth might have yielded a different pattern [56]. Previous work on dental wear among the Hadza residing exclusively in the bush showed extreme sex differences in gross wear on incisors and premolars. Women living only in the bush exhibited significantly more wear than their male counterparts, presumably largely attributed to food processing (peeling tubers with their anterior dentition) [57], although reported sex differences in wear patterning among foraging populations, in general, are equivocal [58].

Strong sex differences in caries prevalence and periodontitis emerged, which mapped onto residence in different ways. When compared to men, Hadza women across all residential settings exhibited the lowest rates of periodontal disease, both in the past and at the time of measurement. Women residing in the bush eating a diet of mostly wild foods exhibited the best oral health overall, exhibiting the lowest rate of caries. Women living in the village consuming a maize dominated diet, however, showed a higher caries prevalence than their female counterparts in the bush or their male counterparts in the village. Hadza men residing in the bush exhibited the worst oral health overall; they exhibited significantly higher rates of broken/missing teeth, higher prevalence of caries, and were significantly more likely to have periodontal disease at the time of study and evidence of it in the past. Interestingly, an intermediate diet composed of both wild foods and domesticated products did not significantly affect caries prevalence for either men or women. This suggests that a diet composed of *primarily* agricultural products appears to present a more cariogenic environment for women, whereas a diet composed of *primarily* wild foods appears to do so for men. These differences may be explained by changes in oral health in a transitioning food economy that more negatively effect women in the village and sex differences in diet composition in the bush that more negatively impact men.

Numerous studies demonstrate that women have significantly higher prevalence rates of caries compared to men across subsistence regimes, both in recent populations [30, 59], including rural Tanzania [60], and archaeological skeletal samples [14, 61, 62] The most oft cited hypotheses for these notable differences are linked to behavior stemming from the sexual division of labor and corresponding dietary differences [30]. More data are needed to determine whether or not Hadza men and women residing in the village have a relatively uniform diet at this point of their transition. It is possible that these sex differences stem from differences in eating frequency (i.e. snacking) [63], or are associated with other factors, such as circulating female hormones and life history events [12]. During menarche and pregnancy, for example, hormonal fluctuations are associated with inflammatory responses that have been shown to negatively impact oral health [64]. It has been suggested that women transitioning from a foraging to a farming economy are at an even *greater* risk for declining oral health. Data from other populations, while limited, suggest that the nutritional transition from foraging to farming may relate to increased fertility and drastic changes in diet, behavior, and salivary and immune health [14].

The most surprising and initially unexpected finding presented here is that Hadza men residing in the bush exhibit the worst oral health. Unfortunately, there are few studies in which to compare our results. The findings that Hadza men in the bush have greater rates of caries support conclusions drawn from earlier work among indigenous Guaraní Indians of Brazil [51], yet are inconsistent with the findings among Aka, Mbuti, and Efe, for which men in the bush had better overall oral health than women [30]. It should be noted, however, that their reported sex differences might be due to the median age of male versus female participants rather than actual sex differences in frequency of carious lesions, or to the fact that women might be more likely to have a greater amount of domesticated cultigens in their diet. We believe that the findings presented here for the Hadza can be explained by sex differences in diet composition in the bush, particularly honey consumption.

Hadza men and women residing in the bush exhibit strong sex differences, not only in foraging behavior [36], but also in food preferences [65] and diet composition [66]. Men have more antemortem tooth loss, possibly due to the fact that they often use their teeth to grip the shaft of arrows when straightening them [57] or due to patterns of honey consumption. Hadza men and juvenile boys living in bush camps routinely target and consume greater amounts of liquid honey from the hives of both stingless and stinging bees compared to women residing in the same camps as [38, 67, 68] as well as their counterparts living in the village, who are reported to consume small amounts of both honey and larvae [40]. The data on the cariogenicity of liquid honey is mixed, with some studies finding that its consumption is linked with the development of caries [69, 70] and some results suggesting that natural honey contains anti-bacterial properties that might act to inhibit bacterial growth leading to caries [71, 72]. While the results are equivocal, it may be that while liquid honey does contain esters that prevent demineralization, they might not override the high sugar content that can lead to caries [73]. Furthermore, it should be noted that as the Hadza consume the entire contents of the hive, the wax from the honeycomb appears to keep the liquid honey affixed to the teeth, effectively marinating the enamel surface in cariogenic sugars and plaque bacteria for hours on end, possibly preventing further food processing from removing them. As noted above, consumption of honey and other sticky, sugar-rich foods has been associated with high caries incidences in prehistoric foragers [23] (although for a notable exception among a living population, the Aka, see [30]).

It is possible that smoking may also contribute to the high caries rate. Estimates suggest that men residing both in the bush and the village have been smoking marijuana for at least two centuries [36]. They have obtained it historically by trading wild game meat or honey with neighboring tribes [74]. They also smoke loose-leaf tobacco, and have been doing so since at least the turn of the 20^th^ century [36, 75]. Over the past decade, however, bush camps have increasingly obtained both tobacco and marijuana. It may be that bush camps have greater sustained access to both, given that tobacco and cannabis can function as payment by tour companies and other visitors to bush camps, although we have no data for smoking behavior in the village with which to compare smoking rates. It is clear, however, that men in the bush smoke both tobacco and marijuana significantly more frequently than do women, although women do chew tobacco on occasion [76]. Smoking tobacco has been shown repeatedly to be a significant risk indicator for periodontal disease and high prevalence of caries [77, 78, 79]. Smoking marijuana has also been shown to positively correlate with high prevalence of carious lesions and periodontitis [80, 81, 82], yet the causal link remains unclear [83]. Future work will aim to quantify the smoking behaviors of individuals living in both the bush and the village to determine if any links can be found between residential location and access to tobacco and marijuana.

The low rates of periodontal disease and caries in women residing in the bush may also be associated with dietary differences. Hadza women consume significantly more tubers than their male counterparts [61]. While tubers are a major source of carbohydrates in the diet [36], they also contain a significant amount of grit and fiber [84, 85], which may act to abrade the bacteria-rich biofilm responsible for caries formation, effectively cleansing the teeth with each chewing bout. Indeed, a negative correlation between frequency of occlusal surface caries and gross tooth wear has been suggested before [9]. Wear obliterates fissures on crowns, limiting areas wherein plaque can accumulate [19, 86]. On the other hand, caries rate has been shown to correlate positively with wear in some samples [87], and, more to the point for this study, there were no differences in gross wear between the sexes. Alternatively, differences might be explained by variation the type of carbohydrate consumed. Just as the introduction of maize during the Neolithic transition in the New World had a greater effect on caries incidence [3, 20, 21] than did wheat in the Near East [22, 23] or rice in East Asia [24], perhaps a village diet dominated by maize-based ugali porridge is more cariogenic than a bush diet rich in tubers.

Another possible contributing factor to the low incidence of caries among women consuming a low agricultural diet may be sex differences in oral microbiome, which have been linked to diet. It has been proposed that Hadza women house bacterial species in their gut that allow them to more efficiently digest tubers [88], differentially adapting them to their diet. This could mean that sex differences in the oral microbiome also allow women to more efficiently break down carbohydrates at the start of digestion, in the mouth, which might have implications for cariogenicity of their oral environment. The oral cavity is a unique and dynamic ecosystem that contains multiple microbial habitats (e.g. teeth, gingival tissue, tongue, cheeks, and lips), all of which are colonized by distinct bacterial communities [5]. Recent work on the composition of microorganisms on the intestinal epithelial interface (the so-called enterocyte associated microbiome) among the Hadza revealed a greater abundance of certain bacterial species compared to Western counterparts [89]. These included species from the genera *Olsenella* and *Slackia*, which are typically associated with caries and periodontitis [90], yet have also been associated with the production of equol, a beneficial phytoestrogen [91], which is linked with positive health benefits like a reduction in rates of cardiovascular disease, some cancers, and osteoporosis [92, 93, 94]. Future work will explore the association between oral bacteria, gut bacteria, diet composition, and dental health among the Hadza consuming wild diets and those dominated by agricultural products.

It should be noted that some Hadza, residing in both the bush and the village, anecdotally report using small branches from the *Salvadora persica* plant to brush their teeth. The plant has antimicrobial properties that evidently defend against some types of oral pathogens [95, 96], gingival irritation [97], and caries formation [98]. We have no data on patterns of use, stratified by age, sex, diet, or residential setting. Future work aims to document such patterns and map them onto prevalence of periodontitis and caries formation.

Limitations of this study include small sample size and the inability to quantitatively document diet composition throughout the life course. As periodontitis and caries prevalence are associated with lifetime diet [99, 100] our interpretations of the relationships between oral health and diet are only as reliable as reports of the subsistence and residency patterns by each individual subject. In addition, all Hadza (regardless of where they are living) consume at least a small percentage of agricultural products. Despite these limitations, however, significances of results parsed by diet and sex speak directly to the efficacy of our locale categorizations as a long-term general diet proxy. Further, we feel that data such as these are critical to better understand the myriad health effects associated with undergoing nutrition transition.

In conclusion, this study, the first to report oral health and prevalence of caries and periodontal disease among the Hadza, supports findings in the burgeoning literature that suggest that foragers do not always have superior oral health when compared to agriculturalists. These data reinforce the notion that the mechanisms of cariogenesis are dependent on a suite of factors that may include diet, behavior, and the oral microbiome. Future work among the Hadza will aim to tease apart such connections.

## Supporting information

S1 Text Diet composition. (DOC)

S2 Text Code sheet for interviews and oral health measures. (DOC)

S1 Table Residence interviews and oral health measures. (CSV)
