# Supplementary material for: Oral health in transition: The Hadza foragers of Tanzania
Source: PLoS One. 2017 Mar 15;12(3):e0172197. doi: 10.1371/journal.pone.0172197 (PMC5351833; doi:10.1371/journal.pone.0172197)
Supplement: S1 Text — (DOC) [file pone.0172197.s001.doc]

**Oral Health among the Hadza: Electronic Supplementary Material**

**Authors:** Alyssa N. Crittenden^a^, John Sorrentino^b^, Sheniz A. Moonie^c^, Mika Peterson^d^, Audax Mabulla^e^, and Peter S. Ungar^f1^

**Supplementary Information: Diet Composition**

*Bush Diet*

Tubers, or underground storage organs, are a staple food of the Hadza diet (1, 2). Species targeted include “matukwayako” (*Coccinea surantiaca*), “matalitako” (*Eminia entennulifa*), “//ekwa” (*Vigna frutescens*), “shakeako” (*Vigna macrorhyncha*), “panjuko” (*Ipomoea transvaalensis*), and “shumuako” (*Vatoraea pseudolablab*). The majority of tubers collected are located up to six feet below the ground surface and are accessed using the sharpened tip of a digging stick. Once tubers are extracted, they are consumed either raw or after briefly roasting on an open fire. All tuber species that have been analyzed are low in fat and protein and relatively high in fiber and simple carbohydrates (2,3).

Baobab fruit (*Adansonia digitata*), or “ngobabe”, is consumed throughout the year, during both the wet and dry seasons. The fruit has an inedible hard outer shell containing approximately 15 – 20 seeds covered with dry pulp that is consumed in several ways (35): (1) directly out of the shell, discarding the hard seed inside the pulp, (2) as flour pounded with seed husks winnowed on the surface of a small piece of animal hide, (3) as a sweet paste formed by combining the flour with water and/or berry juice, or (4) as flour formed by pounding washed and sun-dried seeds removed from the dung of baboons. The fruit pulp is low in fat, protein, and fiber and the pulp flour (seed and pulp combined) is high in fat, protein, and fiber – and both the pulp and flour are high in simple carbohydrates (2).

Berries, figs, drupes, and legumes compose the remainder of the plant-based diet. Berry species targeted include: “undushabe” (*Cordia senensis*), “masakapi” (*Cordia crenata*), “hlukayebe” (*Grewia villosa*), “kongolobi” (*Grewia bicolor*), “nguilabe” (*Grewia ectosicarpa*), and “tafabe” (*Salvadora persica*). All of the berries consumed by the Hadza have large seeds with very little pulp, and when consumed, the seeds are either expectorated or passed through the digestive system with little or no mastication. All berries are low in fat and protein, but high in simple carbohydrates (2). Figs (*Ficus sycomorus*), or “ogoyo”, are a highly desirable Hadza food, targeted mainly by children (4). They are consumed whole, including the small seeds inside of the pulpy fruit, and are low in protein and high in fat, simple carbohydrates, and fiber (2). Two species of drupe are consumed, “pawe” (*Sclerocarya birrea*), which is also known as marula nut, and “mashalobe” (species unknown); both contain a very hard seed surrounded by dense pulp. Marula nut is high in protein and fat and “mashalobe” is low in fat and protein and high in fiber and simple carbohydrates. The only legume species consumed is “mangwala” (*Acacia nilotica*), which is high in protein and fiber and low in fat and simple carbohydrates (3,4).

Honeycomb (including liquid honey and larvae) is ranked as the favorite food item of adults and children (5). The Hadza target the hives of both stinging bees, “ba’alako” (*Apis melllifera*), and stingless bees, “kanoa” (*Trigona rispolii*), “tsunako” (*Trigona gribodoi*), “!nateko” (*Trigona erythra junodi*), “mulangeko” (*Trigona beccarii*), “lulindi” (*Trigona denoiti*), and “bambahau” (*Lestrimellitta cubiceps*). Honey is a concentrated source of fructose and glucose that contains approximately 80–95% sugar (6, 7) and trace amounts of several essential vitamins and minerals (8). Bee larvae is a good source of protein, fat, and several essential minerals and B vitamins (9), and foragers often chew on the entire contents of the hive – including the wax (10).

Birds and small, medium, and large mammals are hunted with bows and arrows throughout the year and targeted almost exclusively by men. Game meat is more abundant during the dry season when animals and people congregate around the limited watering holes (11, 12). The Hadza consume more than 700 bird species (13) and every game mammal that lives in the Lake Eyasi basin, including aardvark, bushbuck, cape buffalo, dik dik, eland, gazelle, giraffe, hyrax, impala, kudu, warthog, wildebeest, and zebra. For an exhaustive list of species targeted, see Marlowe (36) and Peterson et al. (14). Meat is shared widely, both within and outside of the household (15, 16). Wild game meat, when compared to meat from domesticated animals, is lower in saturated fat, and provides moderate to high protein and higher amounts of mono- and polyunsaturated fatty acids (17,18). Sex differences in foods targeted and overall diet composition have been documented extensively among the bush dwelling Hadza. Women, who forage in groups, focus their collection efforts exclusively on plant foods (19) and, as a result, consume a diet that is more heavily based on plants (20, 21, 22). Men, alternatively, focus primarily their collection efforts on game meat and honey (although they do sometimes collect baobab fruit) and tend to go on forays alone or in pairs. They consume much greater amounts of their targeted foods both in and out of camp (11, 20).

**References**

1. Marlowe FW, Berbesque JC. Tubers as fallback foods and their impact on Hadza hunter‐gatherers. Am J Phys Anthropol. 2009; 140(4): 751-758.

2. Schoeninger MJ, Bunn HT, Murray SS, Marlett JA. Composition of tubers used by Hadza foragers of Tanzania. J Food Comp Anal. 2001; 14(1): 15-25.

3. Crittenden AN. Allomaternal care and juvenile foraging among the Hadza: Implications for the evolution of cooperative breeding in humans. Doctoral dissertation, University of California, San Diego. 2009. (Available: <https://escholarship.org/uc/item/27f031k9>).

4. Crittenden AN, Conklin-Brittain NL, Zes DA, Schoeninger MJ, Marlowe FW. Juvenile foraging among the Hadza: Implications for human life history. Evol Hum Behav. 2013; 34(4): 299-304.

5. Berbesque JC, Marlowe FW. Sex differences in food preferences of Hadza hunter-gatherers. Evol Psychol. 2009;*7*(4): 601-616.

6. Bogdanov S, Jurendic T, Sieber R, Gallmann P. Honey for nutrition and health: A review. J Am Coll Nutr. 2008; 27(6): 677-689.

7. Murray SS, Schoeninger MJ, Bunn HT, Pickering TR, Marlett JA. Nutritional composition of some wild plant foods and honey used by Hadza foragers of Tanzania. J Food Comp Anal. 2001; 14(1): 3-13.

8. Alvarez-Suarez JM, Tulipani S, Romandini S, Bertoli E, Battino M. Contribution of honey in nutrition and human health: a review. Med J Nutrition Metab. 2010; 3(1):15-23.

9. Finke MD. Nutrient composition of bee brood and its potential as human food. Ecol Food Nutr. 2005; 44(4): 257-270.

10. Crittenden AN. The importance of honey consumption in human evolution. Food and Foodways. 2001; 19(4): 257-273.

11. Berbesque JC, Wood BM, Crittenden AN, Mabulla A, Marlowe FW. Eat first, share later: Hadza hunter–gatherer men consume more while foraging than in central places. Evol Hum Behav. 2016. (DOI: <http://dx.doi.org/10.1016/j.evolhumbehav.2016.01.003>)

12. Woodburn J. An introduction to Hadza ecology. In Man the hunter. Eds. R.B. Lee and I. DeVore. 1968; pp.49-55.

13. Marlowe FW. Central place provisioning, the Hadza as an example. In: Feeding ecology in apes and other primates. Eds. Hohmann, G., Robbins, M. M., & Boesch, C. Cambridge University Press. 2006;359.

14. Peterson D, Baalow R, Cox J. Hadzabe: By the light of a million fires. Dar es Salaam: Mkuki na Nyota, 2013.

15. Hawkes K, O'Connell JF, Blurton Jones NJ. Hadza meat sharing. Evol Hum Behav. 2001; 22(2): 113-142.

16. Wood BM, Marlowe FW. Household and kin provisioning by Hadza men. Hum Nature. 2013; 24(3): 280-317.

17. Eaton SB, Konner MJ, Cordain L. Diet-dependent acid load, Paleolithic nutrition, and evolutionary health promotion. Am J Clin Nutr. 2010; 91(2): 295-297.

18. Mann N. Dietary lean red meat and human evolution. Eur Jour Nutr. 2000; 39(2): 71-79.

19. Hawkes K, O'Connell JF, Blurton Jones NG. Hadza women's time allocation, offspring provisioning, and the evolution of long postmenopausal life spans. Curr Anthropol. 1997; 38(4): 551-577.

20. Berbesque JC, Marlowe FW, Crittenden AN. Sex differences in Hadza eating frequency by food type. Am J Hum Biol. 2011; 23(3): 339-345.

21. Berbesque JC, Marlowe FW, Pawn I, Thompson P, Johnson G, Mabulla A. Sex differences in Hadza dental wear patterns. Hum Natur. 2012; *23*(3): 270-282.

22. Schnorr SL, Candela M, Rampelli S, Centanni M, Consolandi C, Basaglia G, Turroni S, Biagi E, Peano C, Severgnini M, Fiori J, Gotti R, De Bellis G, Luiselli D, Brigidi P, Mabulla A, Marlowe FW, Henry AG, Crittenden AN. Gut microbiome of Hadza hunter-gatherers. Nat Commun. 2014; 5(3654) doi:10.1038/ncomms4654.
