# Supplementary material for: Oral health in transition: The Hadza foragers of Tanzania
Source: PLoS One. 2017 Mar 15;12(3):e0172197. doi: 10.1371/journal.pone.0172197 (PMC5351833; doi:10.1371/journal.pone.0172197)
Supplement: S2 Text — (DOC) [file pone.0172197.s002.doc]

**Code sheet for interviews and oral health. Details on the oral health variables are presented in the methods section of the paper.**

**Question 2:** The location of the camp where the individual was born.

**Question 3:** The residence location reported by the individual that characterizes the majority of their childhood: bush, village, or both.

**Question 4:** The diet that the individual reported as being the primary diet during the majority of their childhood: bush, village, or both.

**Question 5:** The residence location reported by the individual that characterizes the majority of their adult life: bush, village, or both.

**Question 6:** The diet that the individual reported as being the primary diet during the majority of their adult life: bush, village, or both.

**Question 7:** The reported answer as to whether or not they go to the village: yes, no, or sometimes.

**Categorization of camp**: Category of camp where individual was residing. The category of “bush” is a proxy for a diet low in agricultural products; the category “intermediate” is a proxy for a diet that is variable in agricultural products (with an intermediate proportion of cultigens over a long timeframe); and the category “village” is a proxy for a diet that is high in agricultural products.

**Age:** Age of individual. Estimates of age were determined using long-term demographic data collected over the last 10 years by author ANC and over the last 40 years by anthropologists Frank Marlowe and Nicholas Blurton Jones. Ages of unknown individuals were estimated by ANC based on interview data of camp members and relative age to known individuals from the census.

**Sex:** Sex of individual; m = male and f = female.

**Grosswear:** Gross wear of upper first and second molars was scored as (0) unworn, (1) facet development with at most slight dentin exposure, and (2) a flat occlusal surface with extreme enamel loss and dentin exposure.

**CrowdMeasure:** Anterior dental crowding was measured from photographs as the ratio of the summed mesodistal lengths of the biting surfaces of all lower anterior teeth dived by the straight line distances between distal edges of right and left canines.

**PocketDepth:** The summed depth in millimeters of the six periodontal recesses (mesiobuccal, buccal, distobuccal, mesiolingual, lingual, and distolingual) around the C_1_ and M_1_ of each individual.

**CariesAll%:** The total number of carious cheek teeth, both those with visible caries and those with incipient ones detected only by laser fluorescence, divided by the total number of cheek teeth in the mouth.

**CariesVis:** The total number of cheek teeth with visually identifiable caries divided by total number of cheek teeth in the mouth.

**PerioPast:** Evidence for (1) mild, (2) moderate, and (3) severe periodontitis in the past.

**PerioNow:** Evidence for (1) mild, (2) moderate, and (3) severe periodontitis in the present.

**M3Occlude:** Third molar eruption and occlusion were scored as: (1) no M3s erupted; (2) one or more M3 erupted, but not in occlusion; and (3) all M3s in occlusion.

**Angles:** Occlusion type was scored according to Angle’s classification based on contact location of the M^1^ paracone relative to the buccal groove of M_1_. Scoring indicates (1) neutrocclusion, paracone with the buccal groove, (2) distocclusion, paracone mesial to the buccal groove, and (3) mesiocclusion,paracone distal to the buccal groove.
